# Cellulose Membranes: Synthesis and Applications for Water and Gas Separation and Purification

**DOI:** 10.3390/membranes14070148

**Published:** 2024-06-30

**Authors:** Jinwu Wang, Syed Comail Abbas, Ling Li, Colleen C. Walker, Yonghao Ni, Zhiyong Cai

**Affiliations:** 1 Forest Products Laboratory, U.S. Forest Service, 1 Gifford Pinchot Drive, Madison, WI 53726, USA; 2 Department of Chemical and Biological Engineering, University of Maine, 5737 Jenness Hall, Orono, ME 04469, USA; 3 School of Forest Resources, University of Maine, 5755 Nutting Hall, Orono, ME 04469, USA; 4 Process Development Center, University of Maine, 5737 Jenness Hall, Orono, ME 04469, USA

**Keywords:** cellulose, cellulose derivatives, cellulosic materials, cellulosic membranes, cellulose particles, cellulose nanofibrils, cellulose nanocrystals, nanofibrous membranes

## Abstract

Membranes are a selective barrier that allows certain species (molecules and ions) to pass through while blocking others. Some rely on size exclusion, where larger molecules get stuck while smaller ones permeate through. Others use differences in charge or polarity to attract and repel specific species. Membranes can purify air and water by allowing only air and water molecules to pass through, while preventing contaminants such as microorganisms and particles, or to separate a target gas or vapor, such as H_2_ and CO_2_, from other gases. The higher the flux and selectivity, the better a material is for membranes. The desirable performance can be tuned through material type (polymers, ceramics, and biobased materials), microstructure (porosity and tortuosity), and surface chemistry. Most membranes are made from plastic from petroleum-based resources, contributing to global climate change and plastic pollution. Cellulose can be an alternative sustainable resource for making renewable membranes. Cellulose exists in plant cell walls as natural fibers, which can be broken down into smaller components such as cellulose fibrils, nanofibrils, nanocrystals, and cellulose macromolecules through mechanical and chemical processing. Membranes made from reassembling these particles and molecules have variable pore architecture, porosity, and separation properties and, therefore, have a wide range of applications in nano-, micro-, and ultrafiltration and forward osmosis. Despite their advantages, cellulose membranes face some challenges. Improving the selectivity of membranes for specific molecules often comes at the expense of permeability. The stability of cellulose membranes in harsh environments or under continuous operation needs further improvement. Research is ongoing to address these challenges and develop advanced cellulose membranes with enhanced performance. This article reviews the microstructures, fabrication methods, and potential applications of cellulose membranes, providing some critical insights into processing–structure–property relationships for current state-of-the-art cellulosic membranes that could be used to improve their performance.

## 1. Membranes: Requirements, Types, Classification, and Separation Mechanisms

### 1.1. Requirements of Membranes

A membrane is a thin, pliable sheet of material that allows some components of a mixture to pass through while preventing others based on size, charge, affinity, and other interactions between the permeants and membrane. Membrane technology utilizes this selective permeation to regulate the flow of particles, molecules, and ions for containment, concentration, separation, and/or purification. This technology can improve the process and energy efficiency in the fields of drug delivery, tissue engineering, water filtration, chemical recovery, greenhouse gas capture, moisture removal, etc.

Membranes for commercial applications require high performance, high durability, low cost, and positive environmental impact. The high performance of a membrane can be described as high selectivity and flux (or permeance normalized to the pressure drop and permeability normalized both to the pressure drop and thickness) through the membrane. Selectivity is the ability that allows the desired components to pass through while remaining impermeable to others, which can be calculated in three different ways using component concentrations in the feed and permeate as well as the permeability of components in the membrane, and it is expressed by the retention rate, separation factor, and selectivity coefficient [1]. The selectivity coefficient is defined as the ratio of permeability coefficients of a pair of substances and can be used to characterize membrane materials’ properties. The other two quantities are used to characterize the efficiency of separation processes. Permeability facilitates the efficient permeation of desired components through the membrane. Durability is the ability to resist degradation chemically, biologically, and thermally and fouling from feed mixtures and operating conditions; high durability means less brittleness for easy handling, low susceptibility to swelling and pressure compaction, no detectable aging, and mechanical properties for resisting operation pressures. The membrane system should be scalable for industrial-scale applications. Membrane separation can be more expensive than some traditional separation methods due to the cost of developing and maintaining high-performance membranes.

Membranes are made from a variety of materials, including synthetic organic polymers, inorganic ceramics, and biological substances. Commonly used polymer membranes for water purification include polyamide, cellulose acetate, polyether sulfone, polyvinylidene fluoride, and polyacrylonitrile, known for their designable porosity, superior mechanical, thermal, chemical, and corrosion resistance, as well as their cost-effectiveness [2]. However, these membranes are susceptible to fouling because of their surface hydrophobicity, leading to notable decreases in separation efficiency, flux, and lifespan when treating highly contaminated water sources [3,4]. Swelling due to plasticization and compaction due to creep are two issues for polymer membranes. To address this fouling issue, a frequently employed strategy is to either modify hydrophobic membrane surfaces to enhance their hydrophilicity or replace them with hydrophilic membranes. Furthermore, the extensive use of fossil-based polymers in membrane production has raised sustainability concerns due to the depletion of fossil resources and environmental impacts.

Some inorganic materials include zeolites, silica, perovskites, and metal–organic frameworks. Inorganic membranes generally exhibit far better chemical, mechanical, thermal, and pressure stability than polymer membranes. On the other hand, the application of inorganic membranes is constrained not only by their fragility but also by the elevated operating expenses associated with them [5]. Consequently, researchers have made efforts on the development of environmentally friendly membranes derived from renewable natural resources. These efforts have focused on developing renewable membranes that possess antifouling properties with a hydrophilic nature, along with suitable porosity and necessary mechanical strength [6,7,8].

### 1.2. Types and Classification

**Figure 1:**
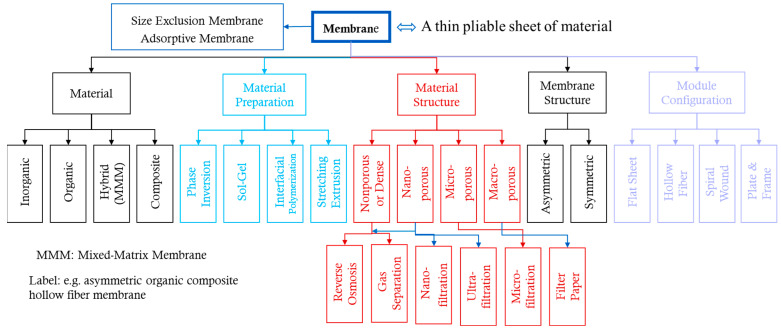
Classification of membranes.

Membranes can be classified based on driving forces, membrane structure, membrane materials, the membrane material preparation method, and membrane materials’ structure (Figure 1). The main driving forces are pressure gradients, concentration gradients, or electrical potential differences such as in lithium battery separation membranes. Based on membrane structure, membranes can be classified as symmetric/asymmetric. Asymmetric membranes have a finger-like pore structure with the sizes of pores gradually changing from one side to the other, and symmetric membranes often have a sponge-like pore structure with relatively monodisperse pores. According to the chemical nature of membrane materials, they can be divided into organic (polymeric), inorganic (ceramic, metallic, glass, zeolite, and carbon), and composites if made from different materials of the same kind or as hybrids made from both organic and inorganic materials. The most common types of organic membranes are cellulose, polyamides, polysulfones, polyethylene, and polycarbonate [1]. Asymmetric polymeric composite membranes comprise a top layer of a nonporous selective barrier, a porous sublayer from a different material that provides mechanical support, and sometimes a backing bottom layer. A hybrid membrane may be made from an inorganic particle-reinforced polymeric material. There are several membrane material preparation methods, such as phase inversion, sol-gel, extrusion, stretching, and interfacial polymerization. According to the membrane configuration, form, and arrangement, they are classified as flat sheet, hollow fiber, spiral wound, or plate and frame membranes [9]. The chosen configuration (spiral wound, tubular hollow fibers, or others) significantly impacts factors like the packing density, pressure handling, and cleaning requirements.

According to IUPAC, a mesopore is defined as a pore with a diameter of 2–50 nm and is intermediate between micropores (≤2 nm) typical of zeolites and macropores (≥50 nm) typical of porous glass. Historically, different fields have their own traditional size classification and naming schemes. The size range for each category of membranes is notional and not strictly defined or consistent in the various studies in the literature. The size classification in this paper is based on the recognized definition of nanotechnology as the manipulation of matter with at least one dimension sized from 1 to 100 nanometers, qualified as nanoporous; less than 1 nm at molecular levels is qualified as nonporous or dense; above 100 nm–10 µm as microporous; and >10 µm as macroporous. Table 1 summarizes size ranges and terms used in this review.

Based on membrane material’s structure and selectivity, membranes can be classified into different types.

Nonporous, dense, or semi-permeable membranes do not have permanent pores and rely solely on fluctuating free volume spaces between molecules and depend on the solution–diffusion mechanism for gas and vapor permeation. Reverse osmosis (RO) and gas separation membranes (the selective layer) are predominantly made from nonporous polymeric materials. RO membranes are designed to be highly selective toward water while rejecting most dissolved salts and other solutes. Gas separation membranes, however, can be tailored to selectively permeate specific gases based on their size, solubility, and other properties.

Nanoporous membranes are a specific type of membrane characterized by the presence of pores in the nanometer range (1–100 nanometers). These pores play a crucial role in enabling various filtration separation processes.

Nanofiltration membranes with pores less than 1–2 nm, typical of zeolites, where Knudsen and surface diffusion occur, can retain ion hydrates and organic molecules for water purification or larger gas molecules for gas separation [1,10,11].

Ultrafiltration membranes with 2 to 100 nm pores can separate larger molecules like proteins and viruses.

Microporous or microfiltration membranes contain microscopic pores ranging from 100 nm to 10 μm that allow molecules to pass while excluding bacteria and fine particulates.

Macroporous membranes or conventional filters contain pores larger than 10 μm, removing yeast cells, coarse particulates, and sand.

### 1.3. Molecules and Particle Separation Mechanisms

Membranes can employ various separation mechanisms based on the differing interactions between the components of a mixture and the membrane. Size exclusion membranes have pores or openings of a specific size that only allow smaller particles to pass through, while larger particles are retained. Adsorptive membranes selectively bind specific components of a mixture onto their surface based on their surface chemical functionality while allowing others to pass through. Depending on specific interactions, absorptive membranes can also be specifically named affinity, ion exchange, chelating membranes, or membrane adsorbents; adsorptive membrane materials usually contain functional groups such as amino, carboxyl, hydroxyl, and sulfonic groups. Adsorptive membranes initially may provide a large capacity of separation without precise control of pore sizes, but their capability decreases with operation time. They need to be regularly regenerated to remove adsorbed species. The terms “filter” and “sieve” are often associated with size exclusion membranes. “Membrane” encompasses a wider range of separation mechanisms beyond just size exclusion. Size exclusion refers to a mechanism for separation filtration when molecules or particles with a size larger than the pore opening cannot diffuse through that pore. Even if the size of a molecule is smaller than the pores, their differential transport rates in the pores can cause the separation of the constituents of a mixture.

Molecules pass through pores in one or a combination of two modes: bulk flow under pressure gradients or external forces and diffusion under concentration gradients. Bulk flow involves the bulk movement of a fluid through interconnected pores within a material. It can be further categorized into viscous flow and capillary flow. Viscous flow is a typical bulk flow under pressure gradients observed in fluids with considerable viscosity when the size of the interconnected pores is relatively large compared to the size of the molecules. Molecules in a viscous flow collide with each other more than they interact with the pore walls. Viscous flow does not provide separation. Capillary flow refers to the spontaneous movement of a liquid through narrow channels or porous materials under capillary forces due to surface tension and adhesive forces. Capillary flow occurs when surface tension and adhesion (interface forces) dominate over bulk forces like hydrostatic pressure and viscosity, resulting in a behavior that deviates from viscous flow due to these interactions. In a porous material, capillary forces can act differently on the two immiscible components of a feed mixture. The component with better wetting characteristics (lower surface tension and stronger adhesion to the solid surface) will be more readily drawn into the pores due to stronger capillary action. The lower wetting component will be less affected by the capillary forces and might remain outside the pores or be displaced by the wetting component. This differential wettability of pore surfaces influences the distribution and transport of constituents in pores, providing the separation of constituents. For example, a membrane that is hydrophobic will allow oil to pass but provide a capillary barrier to the passage of water [12]. The transition from viscous flow to capillary flow is related to the interplay between viscous forces and capillary forces concerning pore size, external pressure, fluid viscosity, and surface tension. A rule of thumb says that flow in porous media is the capillary flow when the capillary number is less than 10^−5^ [13].

Diffusion refers to the movement of individual fluid molecules through the material due to random thermal motion under concentration gradients. Unlike bulk flow, diffusion does not involve bulk movement of the fluid. The size ratio of pores and diffusing molecules and surface chemistry dictate diffusion mechanisms, which can be unique or a combination of molecular diffusion, Knudsen diffusion, configurational diffusion, and surface diffusion [14]. Molecular diffusion is the most common type of diffusion observed in larger pores like that in a non-confined space. Fluid molecules move due to random collisions with each other more likely than collisions with pore walls, following a concentration gradient from high to low concentration zones. This mode rarely provides separation. Knudsen diffusion becomes dominant in small pores where the pore size is comparable to or smaller than the mean free path of diffusing molecules, resulting in more frequent interactions with pore walls than among fluid molecules. Because of the differential mean free paths of constituent molecules in a mixture, Knudsen diffusion may result in the preferential transport of one component and thus separation. Configurational diffusion becomes substantial when the size of the diffusing molecule is comparable to the size of the pores. The molecule may find it difficult to navigate the narrow channels within the pores and instead move along the pore walls or by squeezing through constricted sections. Configurational diffusion allows compatible-sized molecules to pass but blocks larger molecules. This is an important mechanism of separation when the differences in the sizes of constituent molecules are not large. Surface diffusion refers to the movement of adsorbed molecules along the surface of a solid material. Adsorbed molecules are those that cling to the surface due to weak attractive forces. While surface diffusion can occur in all pore sizes, it might become more significant for smaller pores or larger molecules that struggle with molecular diffusion through the pores [1]. This mode becomes significant when the pore size is in the order of 0.6 nm, which is comparable to or slightly larger than the sizes of most gases and water molecules [15]. As a result, molecules interacting with the pore surface (but not too strong to be immobilized as in absorptive membranes) may exhibit enhanced surface diffusion compared to other molecules, leading to preferential mass transfer and separation.

In dense or nonporous polymer membranes, there exist no permanent pore networks but dynamic free-volume spaces. Molecular transport is possible only if a molecule dissolves in the membrane (solubility) and diffuses when local concentration gradients and passages emerge due to the polymer chains’ collaborative movement (diffusivity). This solution–diffusion model is relevant for molecules or ions that are small enough to fit within the free volume spaces of the polymer. Separation occurs due to differences in molecule solubility and diffusivity, two factors in play dictating permeability. When polymer membranes possess a pore size in the range of gas and vapor molecular dimensions, they can be used to separate the mixture of gases and vapors.

In practice, the mode of transport through pores can be a combination of these modes, depending on factors such as pore size, pressure and concentration differences, temperature, and the specific properties of the molecules and the porous material. For example, surface and configurational diffusion may play a significant role in molecular transport in nanoporous materials, especially for smaller molecules, while viscous flow and Knudsen diffusion may dominate in larger pores or at higher pressures. In many cases, the combination of Knudsen diffusion and surface diffusion can lead to preferential mass transfer in porous materials, with smaller molecules diffusing more readily through smaller pores and molecules with stronger surface interactions exhibiting enhanced surface diffusion. Understanding and controlling these mechanisms are important for various applications such as gas separation, filtration, catalysis, and membrane technology.

### 1.4. Factors Affecting Permeation (Permeance and Selectivity)

Membrane gas and vapor separation are used in various applications including the production of nitrogen and oxygen-enriched gases, hydrogen recovery from refinery streams, and CO_2_ separation from natural gas. It is a process that utilizes nonporous membranes such as polyamide and cellulose acetate, or nanofiltration membranes from ceramic materials to separate different gases and vapors based on their molecular size, shape, solubility, and diffusivity properties. The size of a gas or vapor molecule can be estimated by its kinetic diameter (Table 2), which is close to the molecular sieving dimension and is a sensitive measure of the ability to move in pores. Diffusion increases with a decreasing molecule size [16].

Nanofiltration membranes of ceramic, zeolite, and carbon primarily utilize size exclusion as the separation mechanism, which is particularly effective for separating gases and vapors with significant size differences. On the other hand, polymer membranes are modeled as a dense or nonporous material with fluctuating free volume spaces between macromolecules where gases are first dissolved onto the membrane and then diffuse through it at different rates, the so-called solution–diffusion mechanism. Both reverse osmosis and gas separation polymeric membranes rely on the solution–diffusion mechanism for separation.

The volume of a polymer comprises the volume occupied by polymer chains and the free volume between them in the form of “holes”. These spaces are dynamic and constantly fluctuate in size and shape due to the thermal motion of the polymer chains and are crucial for the movement of small molecules, ions, or segments of the polymer chains themselves [19]. Except for the effects of molecular sizes, critical temperature (Table 2) is a measure of the ease of condensation for gaseous molecules, and the solubility increases with it. The membrane material’s affinity for a permeant also plays a role in affecting solution and diffusion. The affinity must not be too strong such that its mobility is reduced. In general, higher solubility in a polymer is obtained for gases with greater critical temperatures and stronger interaction, while a weaker interaction and smaller size of the permeating gas increase diffusion [16]. The gas permeability of a nonporous polymer is controlled neither by the gas diffusivity nor the gas solubility in the polymer but the overall result of diffusivity multiplying solubility [20].

Gaseous substances can be classified as light gases based on molecular sizes or condensable gases such as those with critical temperatures larger than 300 K as well as vapors such as water and volatile hydrocarbons. Condensable gases and vapors typically have high solubility in membranes enabling them to preferentially permeate over the less soluble components including H_2_. Water vapor and CO_2_ have smaller kinetic parameters and higher critical temperatures, typically leading to larger permeation than other gaseous substances. Additionally, CO_2_ is a weak Lewis acid with larger quadrupole moments than other gas molecules; it preferentially adsorbs onto surfaces with Lewis base components because of acid–base interactions. However, strongly adsorbing vapors can clog up the membrane pores. Vapors in a gas mixture affect gas permeation; selectivity typically decreases with an increasing pressure drop across the membrane; the water and other vapors in the membranes may have a plasticizing effect [9]. In summary, pore sizes and pore wall surface functionalities need to be designed for maximal effective and efficient separation.

## 2. Cellulose Membranes

### 2.1. Cellulose Forms

Cellulose is the most abundant biopolymer on Earth. It naturally exists in plant cell walls as a constituent of the wood substance. Pulping and bleaching separate wood cells and removing most lignin and hemicellulose results in cellulose fibers for paper as a feedstock for many other cellulose-based products such as regenerated cellulose, cellulose derivatives, and nanocellulose. Regenerated cellulose is made by dissolving cellulose fibers in a solvent and then consolidating cellulose molecules into films, membranes, or filamentous fibers. Cellulose derivatives are made by reacting hydroxyl groups with various chemicals, resulting in various cellulosic plastics such as water-insoluble cellulose esters like cellulose acetates and cellulose nitrate and water-soluble cellulose ethers like carboxymethyl cellulose (CMC) [21]. Some of these derivatives can be hydrolyzed to remove added functional groups to form regenerated cellulose again. Nanocellulose is the result of breaking down cellulose fiber walls into their constituent nanoscale fibrils or crystals. From cellulose fibers to nanocrystals, there are many possibilities for the degree of breakdown. Typically, they are classified into three categories based on the size of the cross-sectional dimensions: cellulose microfibrils, cellulose nanofibrils (CNFs), and cellulose nanocrystals (CNCs). Cellulose microfibrils are a collection of many small fibril networks, each fibril network originating from a cellulose fiber, breaking down partially but not completely, specially made via mechanical refining. CNCs are individualized acicular-shaped particles. CNFs are ultrathin fibrils with a size between those of microfibrils and CNCs. Traditionally, regenerated cellulose and cellulose derivatives have been used to create fine dialysis membranes. However, newly emerging technologies such as cellulose nanotechnology are being used to make cellulose membranes. The utilization of these membranes for industrial material separation has been hindered by numerous factors, including a low mechanical resistance, high fabrication cost, and issues in scaling up.

### 2.2. Cellulosic Membrane Classification

❖ Cellulose membranes made from the solution of cellulose and cellulose derivatives
  ✓ Regenerated cellulose membranes
  ✓ Cellulose derivative membranes
    ○ Cellulose acetate membranes
    ○ Cellulose nitrate membranes
    ○ Ethyl cellulose membranes
    ○ Composite membranes
❖ Cellulose membranes made from cellulose particles
  ✓ Cellulose fiber filters
  ✓ Electrospun cellulose nanofiber membranes
  ✓ Nanocellulose membranes
    ○ Cellulose nanofibril membranes
    ○ Cellulose nanocrystal membranes
  ✓ Composite membranes
    ○ Cellulose particle-reinforced polymer membranes
    ○ Polymer fiber-reinforced cellulose membranes

### 2.3. Potential of Cellulose as a Material for Membranes

In pursuit of sustainable membrane material development with a focus on high hydrophilicity, ideal porosity, and desirable mechanical strength, cellulose has become a central focus of interest among membrane researchers [22]. This intensified interest stems from a multitude of key advantages, including its intrinsic hydrophilic nature, high crystallinity, insolubility in various solvents, non-toxicity, and ease of processing, all of which are underpinned by the robust hydrogen bonding within cellulose [23]. A particularly notable facet of cellulose-based materials lies in the abundant functional groups, such as hydroxyl and carboxylate groups, present in nanocellulose. These functional groups serve as exceptional adsorption sites, effectively removing numerous organic and inorganic contaminants. Moreover, cellulose contributes to the development of mechanically robust membranes characterized by excellent porosity. In the realm of water filter membranes, cellulose serves versatile roles, functioning as a filler to enhance mechanical strength and reduce pore size distribution, as a barrier layer for selective filtration, as a self-standing membrane for efficient nanoscale filtration, or as a support layer to bolster structural stability and the overall membrane performance [24].

**Figure 2:**
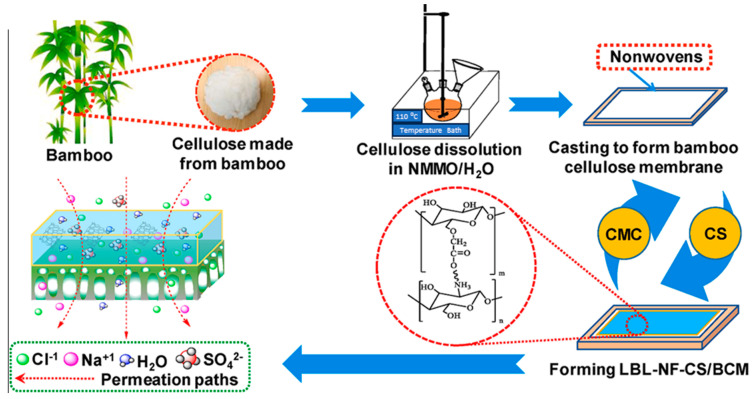
Schematic of cellulose membrane technology from a cellulose source to a filtration application, including the creation of a nanofiltration film on a porous regenerated cellulose-based support (LBL-NF-CS/BCM), adapted with permission from reference [26].

This distinctive amalgamation of properties underscores cellulose as an exceptionally promising and environmentally friendly material for advancing membrane technology, particularly in the pursuit of sustainable solutions for water purification. An illustrative example of this research direction is the work of Li et al., who developed a composite membrane for nanofiltration (NF) labeled as LBL-NF-CS/BCM, comprising a selective film of alternate CMC and chitosan (CS) layers through layer-by-layer spray coating (LBL) on a porous regenerated bamboo cellulose membrane (BCM) as the support (Figure 2) [25]. The cellulose-based composite membrane had an average pore size of 2.2 nm and could be considered a nanofiltration membrane. Under optimized conditions with a transmembrane pressure difference of 0.3 MPa, the membrane had a flux of 12 L m^−2^ h^−1^ and a substantial NaCl rejection rate of 36%.

## 3. Preparation and Structure of Cellulosic Membranes through Dissolution

### 3.1. Phase Inversion

**Figure 3:**
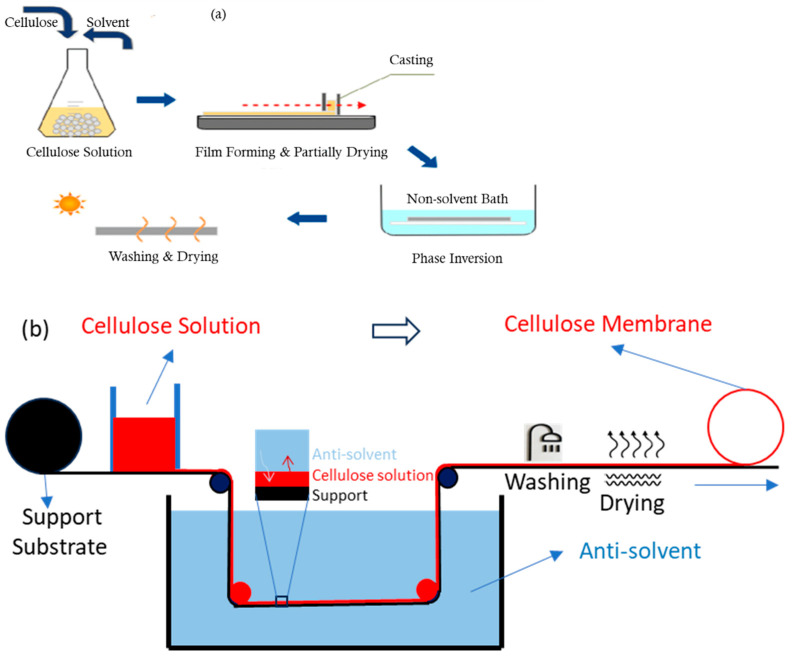
Schematic of making phase inversion membranes: (**a**) lab preparation adapted from [27] and (**b**) a roll-to-roll continuous flat sheet cellulose membrane process, redrawn from [28,29].

Cellulosic membranes made through the solution of cellulose and cellulose derivatives are prepared predominantly via phase inversion, like most other polymeric membrane fabrication methods. Phase inversion is a process of de-mixing a homogeneous polymer solution into a solid phase of the polymer and a liquid phase of the solvent. Various ways to induce this phase separation include cooling, changing pH, adding an anti-solvent, or supersaturation. An anti-solvent is usually introduced to exchange or partially exchange the solvent, causing polymer macromolecule precipitation to form a networked structure. The typical process of making phase inversion membranes is as follows (Figure 3):

Solution preparation: A polymer is dissolved in a suitable solvent to form a homogeneous solution.

Casting: The polymer solution is cast through a slit onto a support flat surface, like a glass plate or a conveyor belt (Figure 3).

Controlled evaporation: The cast film is exposed under programmed temperatures and humidities. If an anti-solvent is already added to the solution, the solvent’s evaporation due to high volatility enriches the anti-solvent and polymer content, leading to partial phase separation.

Immersion: The cast film is immersed in an anti-solvent bath, which induces the formation of a solid membrane by inducing the solvent exchange and phase separation of the solvent and polymer.

Washing: The membrane is washed to remove any residual solvent and anti-solvent.

Drying: The membrane is dried to remove any remaining water or solvent.

### 3.2. Structure of Phase Inversion Membranes

**Figure 4:**
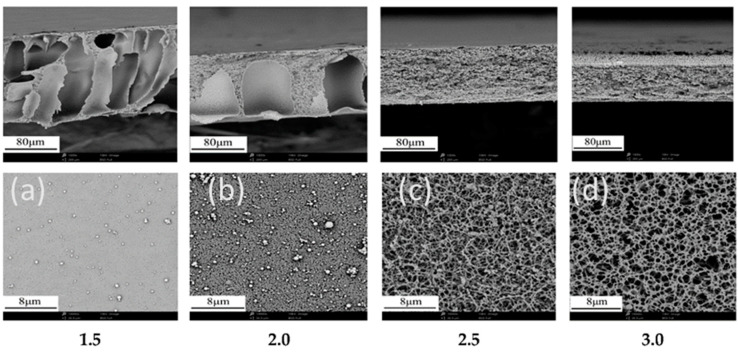
Cross-sections of phase inversion membranes of cellulose acetate (CA): asymmetric structure of dense skin/finger-like pores (**a**,**b**) and homogeneous sponge-like structures (**c**,**d**). The microstructure was tuned with the ratio of the anti-solvent glycerol to CA with acetone/dimethylacetamide as the solvent [30].

When the cast film is added to the anti-solvent bath, the polymer precipitates, and pores can form within the membrane structure. Factors such as the type of polymer, solvent and anti-solvent, polymer concentration, the additives in and temperature of the casting solution and anti-solvent bath can influence the phase separation process and final membrane morphology as well as its separation properties such as permeability, diffusivity, and selectivity [30]. An anti-solvent reduces the ability of a solvent to dissolve a particular solute, causing the solute to precipitate out of the mixture of the solute, solvent, and anti-solvent. A non-solvent is a liquid with little to no ability to dissolve a particular solute, while the term anti-solvent connotes disrupting existing solutions. The degree of miscibility between the solvent and anti-solvent significantly affects the phase separation process and membrane morphology [31,32]. If the anti-solvent is miscible with the solvent, faster exchange between the phases can lead to an asymmetric structure comprising a denser skin layer with smaller pores on top and a more porous sublayer with finger-like pores underneath (Figure 4a,b). The skin layer is responsible for permeability and selectivity, while the sublayer provides mechanical strength and support. These membranes generally have lower permeability due to the denser skin and larger maximum pores. When the cast film is added to the anti-solvent bath, the solvent in the cast film rushing toward the anti-solvent side drags the polymer along with it and meets the anti-solvent moving in reverse at the interface, leading to the formation of two distinct phases: a dense polymer-rich phase that forms the skin layer of the membrane and a lean phase rich in anti-solvent due to the removal of the solvent that forms the sublayer. Pores initiate in the frontier of the anti-solvent and grow into finger-like pores in the lean phase due to polymer coalescence induced by the arriving anti-solvent. When the solvent and anti-solvent are immiscible, limited miscibility restricts the interaction between the two phases, resulting in a slower exchange rate. This slower exchange leads to a “spongier” morphology with smaller, interconnected pores closer to the membrane surface (Figure 4c,d). These pores offer higher permeability but poor selectivity.

The regeneration mechanisms for a cellulose solution in an ionic liquid (1-ethyl-3-methylimidazolium acetate) by anti-solvents of water, methanol, and ethanol are simulated with molecular dynamics under different temperatures and concentrations. The regeneration efficiency sequence is H_2_O > CH_3_OH > CH_3_CH_2_OH, which is the same order of magnitude as of their polarity. Water is the most effective anti-solvent that disrupts the interactions between the cellulose and the ionic liquid, promoting cellulose precipitation and regeneration. Decreasing the temperature and increasing the concentration of anti-solvents will enhance regeneration [33]. Specific disruption mechanisms depend on the interaction of the pair of solvent and anti-solvent. The exact nature of the mixture’s phase separation can vary after adding an anti-solvent. In some cases, a clear separation between solvent, anti-solvent, and cellulose might occur. In other cases, it might be a more complex mixture with interphases. Research is ongoing to develop more efficient and controlled cellulose regeneration processes from solvent and anti-solvent pairs [34].

### 3.3. Factors Affecting Structure and Properties of Phase Inversion Membranes

**Figure 5:**
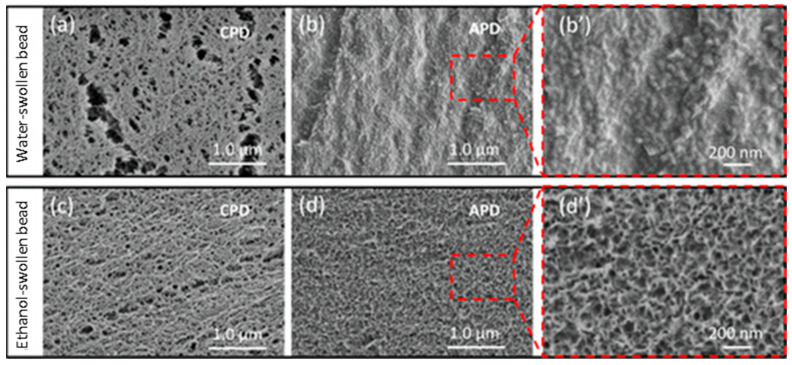
The effect of washing and drying methods on regenerated cellulose membrane structure as revealed by scanning microscopic images. Solvent: lithium chloride/dimethylacetamide; anti-solvent: aqueous ethanol, 96 vol %; washing liquid: Milli-Q water or 96% ethanol; and drying method: supercritical point drying (CPD) (**a**,**c**), or ambient pressure drying (APD) (**b**,**d**); the (**b’**,**d’**) are the amplification of the highlighted square areas in the (**b**,**d**) [35].

It is important to note that miscibility is one factor affecting membrane morphology. Other factors like polymer concentration, coagulation bath temperature, additives, washing liquids, and drying methods also play a role. Figure 5 shows the effect of washing and drying methods on regenerated cellulose structure prepared by phase inversion [35]. This investigation used a drop of a regenerated cellulose solution (cellulose bead) to investigate its structure evolution under different solvent systems, washing, and drying, which reflects the preparation of phase inversion-regenerated cellulose membranes. Hence, these dried bead structures and morphology provide insights into membranes’ structure and morphology. Using cellulose beads is convenient for exploration and imaging in the lab. Supercritical CO_2_ drying generates an open cellulose fibrous network with pores for both water and ethanol-washed and swollen samples, which was deemed as the structure in the wet state before drying since the capillary forces exerted on the cellulose network by the evaporating liquid can nearly be eliminated by supercritical drying. Ambient pressure drying seems to collapse water-washed samples to form a dense structure without visible pores at the designated resolution. Still, it might contain smaller pores (Figure 5b’), but it generates a porous structure for ethanol-washed samples (Figure 5d’). Solution casting without anti-solvent coagulation and drying without constraints, especially in solvents with high surface tension like water, can create a very dense structure.

In summary, combining an understanding of miscibility with these other variables allows for optimizing the phase inversion process for specific needs. Pore size indicates the largest pore diameter and can be related to the membrane’s ability to filter out particles of a certain size. Cellulose membranes can be tuned with a range of pore sizes from hundreds of micrometers to a few nanometers to hundreds of Daltons for the nominal molecular weight cutoff for microfiltration, ultrafiltration, and nanofiltration [36].

**Figure 6:**
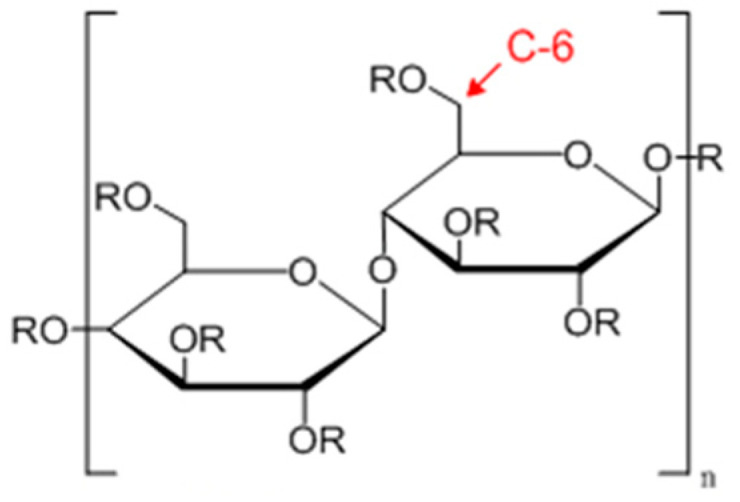
The chemical structure of cellulose (-H), cellulose acetate (-COCH_3_), cellulose nitrate (-NO_2_), carboxymethyl cellulose (-CH_2_COOH), or ethyl cellulose (-CH_2_CH_3_). The red text, C-6, marks the exocyclic carbon in one of the rings, adapted from [37].

To fabricate membranes with the phase inversion method, the polymer must be soluble in a solvent mixture. Phase inversion is often used to fabricate membranes of regenerated cellulose, cellulose acetates, and cellulose nitrates. There are two types of hydroxyl groups in cellulose: ring hydroxyl groups and the C-6 exocyclic hydroxyl (Figure 6). Different reactions occur depending on reagents and reaction conditions, leading to different degrees of the substitution and distribution of derivative groups, such as acetyl, along the cellulose chain. Dissolving cellulose is difficult and needs special solvents. Derivation, such as acetylation and nitration, makes cellulose derivative dissolution easier. The combination of phase separation and mass transfer affects the membrane structure. Their porosity and morphology can be tuned for specific application requirements. They are widely used in various applications, including water purification, filtration, and separation.

### 3.4. Regenerated Cellulose Membranes 

Regenerated cellulose membranes are hydrophilic, spontaneously wet in water, and chemically resistant to removing particles from aqueous and organic solutions. They can be sterilized and have low protein binding and extractables, enabling their use with biological samples. To prepare membranes, dissolving pulp is used, which typically contains >96% cellulose. Traditionally, cellulose membrane is made by converting purified cellulose to a soluble derivative, such as xanthate or cuprammonium cellulose complex, and then precipitating and regenerating from its coagulation bath. Recently, there have been several solvent systems designed to directly dissolve cellulose, such as N-methylmorpholine-N-oxide (NMMO), ionic liquids, LiCl/N, N-dimethylacetamide, NaOH aqueous solution, Alkali/urea and NaOH/thiourea aqueous solution, and Tetra butyl ammonium fluoride/dimethyl sulfoxide systems [34], etc. Anti-solvents are selected depending on their interactions with solvents. A series of anti-solvents for the regeneration of cellulose in NaOH/urea include aqueous solutions of H_2_SO_4_, CH_3_COOH, H_2_SO_4_/Na_2_SO_4_, Na_2_SO_4_, (NH_4_)_2_SO_4_·H_2_O, C_2_H_5_OH, and (CH_3_)_3_CO. Aqueous H_2_SO_4_/Na_2_SO_4_ solutions result in a homogeneous structure with high optical transmittance and mechanical properties. Cellulose regeneration in water generates a pore architecture different from that in alcohols because of the strong polarity and surface tension of water [35].

In tuning various variables, regenerated cellulose membranes can be made in various pore sizes from a few hundred Daltons’ of the cutoff molecular weight to hundreds of micrometers. Most fall in ultrafiltration and nanofiltration for concentrating or desalting dilute solutions. The hydrophilic, tight microstructure of regenerated membranes assures the highest possible retention with the lowest possible adsorption of protein, DNA, or other macromolecules. Using the ionic liquid 1-Allyl-3-methylimidazolium chloride as wood pulp solvent and water as the anti-solvent, regenerated cellulose membranes with pores falling in nanofiltration were developed with an ethanol permeance of around 30 L m^−2^ h^−1^ bar^−1^, outperforming state-of-the-art polymeric membranes. When used for solute separation, a membrane provides molecular selectivity of up to 294 and 68 for Alcian blue/Rifampicin and Alcian blue/Tetracycline mixtures, respectively. The findings reveal the potential of regenerated cellulose as a high-performance and sustainable alternative membrane material for separating organic mixtures [38]. Markarov et al. [39] reported that cellulose membranes obtained from NMMO have a higher permeability to ethanol, a lower rejection of orange II dye, a higher ultimate strength and modulus, and a lower elongation at break than cellophane films with the viscose process. The two types of cellulose membranes differ in structure and morphology: The NMMO process results in a non-uniform porous structure with larger crystallites, a higher degree of cellulose polymerization, orientation, and crystallinity, and plenty of larger anisometric pores than the viscose process, which results in a relatively more homogeneous structure with many smaller pores.

Dense cellophane films can be treated with various aqueous ethanol solutions to tailor their porosity and solvent transport properties across more than two orders of magnitude. Water-solvated ionic dyes have higher rejection rates in these membranes [40]. Mao et al. demonstrated that NMMO cellulose membranes have great potential in the isopropanol dehydration process. For a 95-percent aqueous isopropanol solution, the developed cellulose membrane had a flux of 13.4 g/m^2^·h and a separation factor of 61,271, and water content in the permeate reached 99.97 wt.% [41]. Recent reviews have summarized advances in cellulose dissolution, regeneration, and membrane applications [38,42].

### 3.5. Cellulose Acetate Membranes

Cellulose acetates are produced by reacting cellulose with acetic anhydride, replacing some hydroxyl (OH) groups with acetyl (-CH_3_CO) groups. In what is commonly known as cellulose acetate, no less than 92% of the hydroxyl groups are acetylated. In cellulose triacetate (CTA), only ~1% of the hydroxyl groups remain free, and of these, about 80% are C-6 hydroxyls [37]. Cellulose acetate can be made to be from slightly hydrophilic to highly hydrophobic depending on the presence of remaining hydroxyl (OH) groups from the original cellulose [43]. As the degree of acetylation increases, the overall hydrophobicity of the cellulose acetate also increases. While a moderate level of acetylation can promote crystallinity when combined with controlled heat treatment, it is crucial to consider the interplay of various factors to achieve the desired outcome for specific applications. Cellulose acetates are known for their effective film-forming properties, toughness, and smoothness, which make them suitable for membrane fabrication [44,45]. Hydrophilic cellulose acetate membranes have a very low protein binding capacity and are excellent for protein recovery and osmotically driven membrane processes, but they have limited chemical resistance. A higher degree of substitution yields superior salt rejection but lower flux values due to increasing hydrophobicity [46]. Cellulose acetate membranes are less prone to absorptive fouling than more hydrophobic membranes [37].

Cellulose acetate can be dissolved in organic solvents to form membranes via the phase-inversion process [30]. Wang et al. report a fabrication method that uses a mixture of acetone and dimethylacetamide as the solvent and glycerol and water as the anti-solvents. Through adjusting the ratios of the agents and processing conditions to change the rate of phase separation, the microstructure was tuned from an asymmetric structure of a dense skin/porous underlayer (Figure 4a,b) to a material network with relatively homogeneous pores (Figure 4c,d). The maximum pore size and porosity ranged from 0.61 to 2.33 μm and 85 to 95 percent, respectively, and the water permeance ranged from 182 to 24,472 L^−2^ h^−1^ bar^−1^, falling in the category of microfiltration. The salient influencing factors were the ratios of acetone/dimethylacetamide and glycerol/cellulose acetate, where glycerol was found to perform the dual function of pore formation and plasticization [30]. Glycerol has a high boiling point and interacts with cellulose through hydrogen bonding, making it difficult to completely displace with water or drying, leading to residual glycerol in the polymer that can be beneficial for maintaining the flexibility and processability of cellulose acetate but negatively impacts mechanical properties and thermal stability.

**Figure 7:**
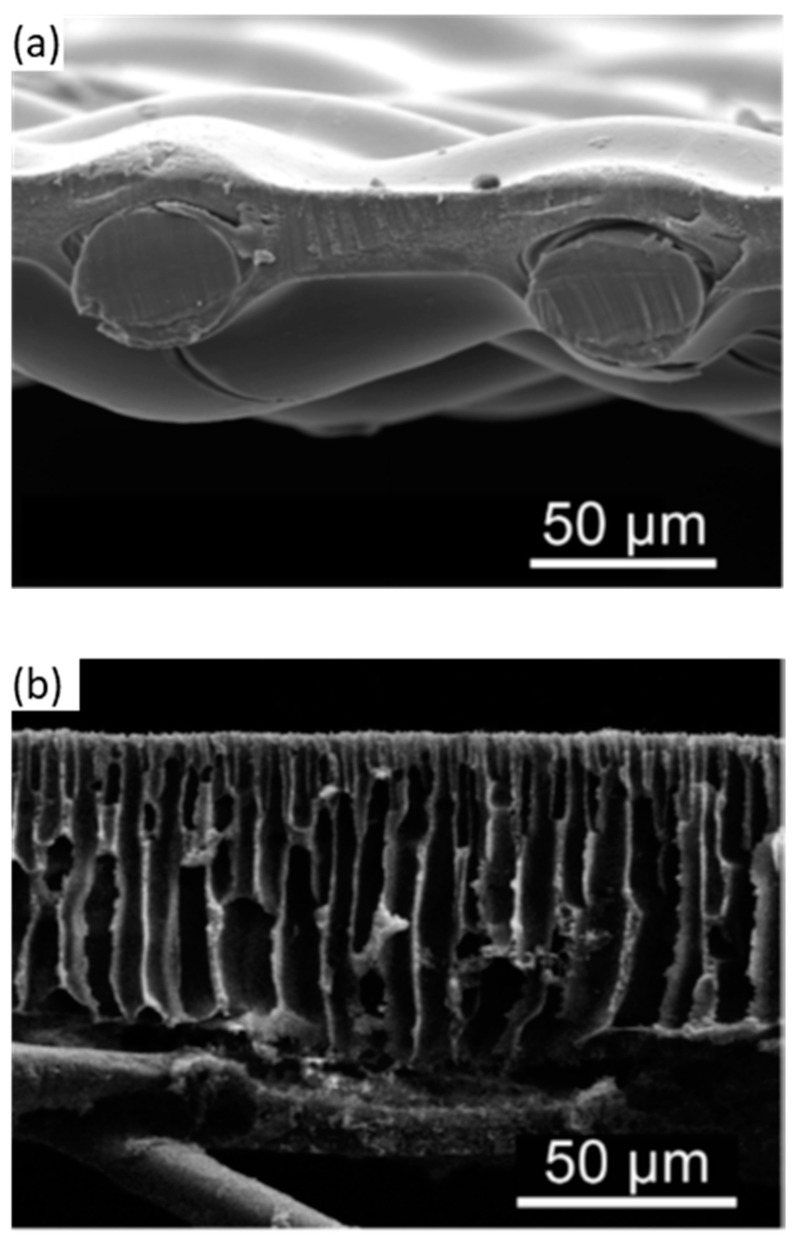
SEM micrographs display the cross-section of (**a**) a commercial HTI-CTA membrane comprising a polyester mesh embedded in cellulose triacetate (CTA) substrate and (**b**) a thin-film composite membrane including a top ultrathin polyamide selective barrier layer, a microporous polysulfone support layer, and a bottom PET nonwoven backing layer [56].

Cellulose acetate membranes can be structured for ultrafiltration, nanofiltration, reverse osmosis, and forward osmosis [47,48,49] in the fields of water purification, protein purification, heavy metal removal, dye wastewater treatment, blood hemodialysis, and oil–water separation [50,51,52,53,54]. Asymmetric cellulose acetate membranes developed in 1959 were the first viable reverse osmosis membranes [9] and dominated the field until the commercialization of thin-film composite membranes in 1972. The first commercially available membrane for osmotically driven processes, developed by Hydration Technologies, Inc. (HTI), consisted of a thin woven polyester mesh embedded within a cellulose triacetate (CTA) substrate (Figure 7a) [55] and exhibited a lower water permeability of 0.36 L m^−2^ h^−1^ atm^−1^ and a salt rejection of 94 percent [56]. A thin-film composite membrane (Figure 7b), including a top ultrathin polyamide selective barrier layer, a microporous polysulfone support layer, and a bottom PET nonwoven backing layer, demonstrated a significantly higher value of 1.16 L m^−2^ h^−1^ atm^−1^ and a slightly higher salt rejection of 97% [57]. The HTI membrane design eliminated the need for a thick backing layer but resulted in a denser membrane that is far thicker than the selective barrier layer of the thin-film composite membrane, while the latter offered superior water permeability, potentially leading to higher water production rates but still maintaining a comparable salt rejection. Instead of polysulfone, cellulose acetate can also be used as the support layer for the polyamide active layer, increasing the number of possible material choices in thin-film composite membranes [57].

However, because of the presence of β-dehydrated glucose, cellulose acetate is susceptible to bacterial attacks, leading to significant damage to the membrane surface and compactness [58]. Cellulose acetate membranes are susceptible to hydrolysis in both alkaline and acidic environments, limiting their operational pH range, typically between pH 4 and 6 [59]. These drawbacks and high fragility have recently prompted a shift toward alternative membrane materials.

To address this challenge, NH_2_-functionalized cellulose acetate and silica composite nanofibrous membranes have been developed to enhance antibacterial properties, which exhibit a remarkable adsorption capacity for Cr^6+^ ions (19.46 mg g^−1^) compared to pure cellulose acetate (1.28 mg g^−1^) or cellulose acetate/silica composites (3.03 mg g^−1^) [60]. Furthermore, cellulose acetate-based membranes have also been employed to remove pharmaceutical and personal care products. For instance, Emam and colleagues established a highly porous photoactive cellulose acetate@Ti-MIL-MOF film, exhibiting both an adsorption and photodegradation capacity for paracetamol in the presence of visible light [61]. The overall paracetamol removal significantly increased from 72.6 mg g^−1^ for the pure Ti-MIL-MOF film and 82.7 for the cellulose acetate film to 519.1 mg/g for porous the CA@Ti-MIL-NH_2_ film. Vatanapour et al. [62] reviewed cellulose acetate applications in the fabrication of separation membranes in detail.

### 3.6. Other Cellulose Derivative Membranes

Cellulose nitrate is a cellulose ester, a derivative of wood pulp. Membranes from cellulose nitrate feature high protein binding, low extractable substances, and a narrow pore size distribution. While cellulose nitrate is often considered to be brittle and thermally unstable, its membranes can be increased in strength, flexibility, and thermal stability to be autoclaved without shrinkage or integrity loss. For example, cellulose nitrate was dissolved in a solvent (acetone) and anti-solvent additive (glycerol) to form a 7 wt.% polymer solution. Phase separation was induced by moisture absorption from the exposed environment, the evaporation of the solvent, and finally, immersion in water to remove the solvent and additive. The prepared membranes had a pore size of up to 8 μm and were applied to adsorbing bovine serum albumin with a maximum adsorption capacity of 1.10 g/m^2^ [63].

Ethyl cellulose is a cellulose ether formed by replacing some hydroxyl groups of cellulose with ethyl groups, typically by reacting cellulose with ethyl chloride. Commercially available ethyl cellulose typically has a degree of substitution between 2.2 and 2.6, making it highly hydrophobic and chemically stable. It also has a film-forming capacity and good mechanical properties for the fabrication of ethyl cellulose membranes [64]. Ethyl cellulose is practically insoluble in most common polar solvents like water, glycerol, and propylene glycol, so they can be used as anti-solvents. However, it becomes soluble in some organic solvents with lower polarity, such as chloroform, toluene, ethanol mixtures, and aromatic hydrocarbon, which can be used as solvents. Table 3 shows the permeability and selectivity for the gas separation of a typical ethyl cellulose and cellulose nitrate membrane.

### 3.7. Composite Cellulosic Membranes

Mixed cellulose ester membranes are composed of more than one cellulose derivative, such as the blend of cellulose acetate and cellulose nitrate as the selective layer. Asymmetric CMC-chitosan polyelectrolyte complex membranes were synthesized by manipulating the pH of the solution and coagulation bath and have the ability as microfiltration membranes to separate oil droplets from emulsions [65]. The composition of regenerated cellulose and cellulose derivatives with wide-length scale inorganic and organic particles and fibers or incorporated with other substances has been investigated in the literature [21]. Their effectiveness and efficiency concerning performance and economic and environmental sustainability depend on their applications [62].

### 3.8. Water Vapor Separation Applications

**Figure 8:**
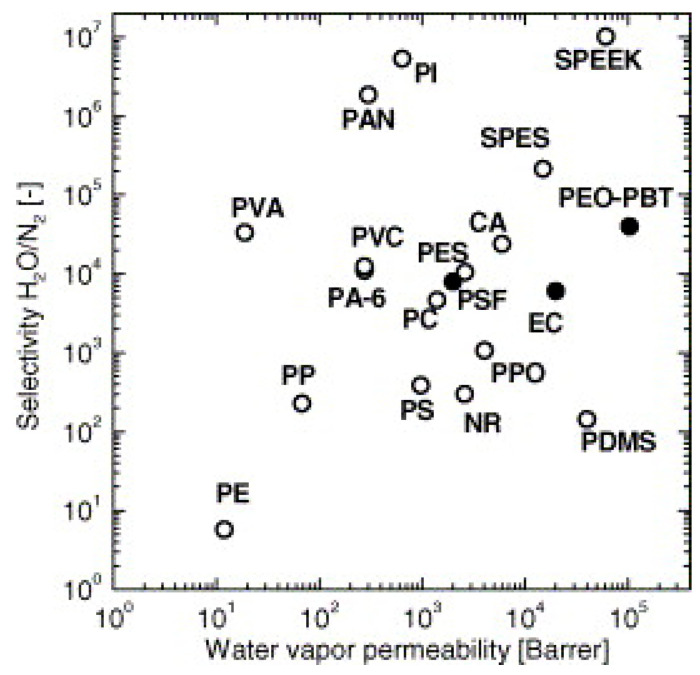
Comparison of water vapor permeability and water vapor/N_2_ selectivity for various polymers at 30 °C [70]. CA: cellulose acetate.

Cellulosic materials generally absorb moisture and are plasticized by it. Their free volume decreases initially and then increases again with increasing humidity depending on the degree of hydroxyl substitution; their gas and vapor permeability coefficients also follow a pattern similar to that of the free volume [66,67]. Table 3 shows that cellulosic membranes preferentially permeate water vapor. They have higher moisture solubility but lower diffusivity than those of high-free-volume polymers such as silicone rubbers. Cellulosic membranes have the potential to be utilized to dehydrate exhaust air to recover water and heat for energy recovery ventilation. There are three typical ways to dry air streams. A refrigerated dryer consuming electricity dehydrates air by cooling it down to condense the water while desiccant dryers dehydrate air by adsorbing water on a solid granular absorbent that needs to be regenerated regularly. In contrast, membrane dehydration provides a wide range of drying capacities depending on membrane separation properties. Cellulosic membranes have higher selectivity coefficients but lower permeability than polydimethylsiloxane (PDMS) membranes. PDMS has high permeability coefficients, owing to its large free volume, and reasonable selectivity for condensable gases, and it has been studied in detail because of the vast utility of PDMS for the removal or harvest of water from air [68,69]. Silicone rubber is extremely permeable and has adequate vapor/inert gas selectivity for most applications; composite membranes of silicone rubber are used in almost all installed vapor separation systems [15]. Compared with other high-performance polymers (Figure 8), cellulose acetate membranes are reasonably performative [9]. Cellulose acetate is an amorphous polymer that lacks a highly ordered crystalline structure. This can benefit both gas and vapor permeability, limiting vapor/gas selectivity. Due to its moderate hydrophilicity, CA exhibits only moderate water permeability.

### 3.9. Carbon Dioxide Capture Applications

Table 3 shows that regenerated cellulose is a good barrier to gases and is poorly selective for different gases in the dry state. It gradually loses its barrier and is increasingly permeable to nitrogen with increasing moisture content. It also can be seen that the structure of regenerated cellulose was prepared to be more porous to increase gas permeability and selectivity, especially in a wet state [71]. Cellulose acetate, initially developed for membrane reverse osmosis, is the most widely used and tested material for CO_2_ removal [15]. Cellulose acetate contains polar functional groups such as carbonyl (C=O) and residual -OH groups, which strongly interact with CO_2_ molecules, affecting absorption and enhancing the CO_2_ permeance. They are used to fabricate large-scale CO_2_ separating membranes [62]. It is noted that cellulose nitrate membranes have relatively lower nitrogen permeability and higher selectivity coefficients toward nitrogen.

Nonporous ethyl cellulose membranes have been used to separate CO_2_ owing to polar ether oxygen atoms that interact with quadrupolar CO_2_ molecules resulting in high solubility [64]. Because of this interaction, it has slightly lower diffusivity in ethyl cellulose than O_2_ even though CO_2_ has a smaller kinetic diameter than O_2_. Solubility is a key factor that enables CO_2_ to preferentially permeate over less soluble gases_._ However, the affinity of membrane materials for CO_2_ must not be too high such that its mobility is reduced; otherwise, the CO_2_ flux will suffer. In general, ethyl cellulose has a larger permeability to CO_2_ than to other light gases, which tends to increase with increasing ethyl content in the polymer, probably due to an increase in the polymer free volume [20]. Compared with cellulose acetate, ethyl cellulose has a higher gas permeability and a lower CO_2_/ N_2_ selectivity because of its larger free volume and higher chain mobility.

**Table 3:** Gas permeability and selectivity of cellulosic membranes at 25 °C and relevant humidity (RH) [72].

Cellulosic Material		Permeability (Barrer)					Selectivity Coefficient			
		H_2_O	CO_2_	H_2_	O_2_	N_2_	H_2_O/N_2_	CO_2_/N_2_	H_2_/N_2_	O_2_/N_2_
Regenerated cellulose	0%RH		0.005	0.006	0.002	0.003		1.5	2.0	0.7
	43%RH		0.013	0.016	0.007	0.007		1.9	2.4	1.1
	76%RH		0.072	0.033	0.009	0.007		9.6	4.4	1.2
	100%RH	25,198	0.256	0.080	0.012	0.018	1,369,565	13.9	4.3	0.6
	Dry *		127.7	9.14	6.28	2.58		49.5	3.5	2.4
	Wet *		1957.4	134.7	93.42	37.13		52.7	3.6	2.5
Cellulose acetate **		7333	23.07	3.506	0.780	0.280	26,191	82.4	12.5	2.8
Cellulose nitrate		6293	2.120	2.000	1.947	0.116	54,253	18.3	17.2	16.8
Ethylcellulose		8933	113.1	87.06	14.67	4.426	2018	25.5	19.7	3.3
Polydimethylsiloxane		43,000	4651.6	939.9	926.6	470.6	91.4	9.9	2.0	2.0

* [71]; ** plasticized.

## 4. Nonwoven Membranes from Cellulose Particles

### 4.1. Types of Cellulose Particles

**Figure 9:**
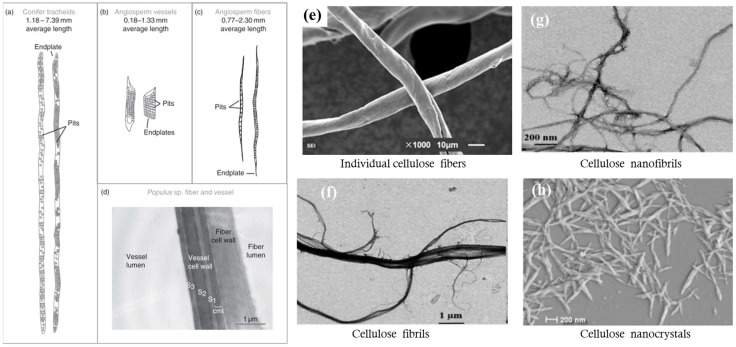
The size and morphology of major wood cells [74]. (**a**) Softwood tracheid, (**b**) hardwood vessels, (**c**) hardwood fibers, (**d**) cell wall structure (S_1_–S_3_) of two neighboring wood cells. Fiber development by refining: (**e**) isolated cotton fibers, also known as cellulose fibers, at zero-fibrillation refining time, showing a complete absence of fibrils, i.e., 0% degree of fibrillation [75], (**f**) part of a broken fiber with attached smaller fibrils or fibrillar fines, (**g**) networked nanofibrils [76], and (**h**) cellulose nanocrystals formed via acid hydrolysis of cellulose fibers [77].

Cellulose exists mostly in plant cell walls such as wood, cotton, flax, and bamboo, and it is also produced by different types of bacteria and animals. Predominant wood cells are tracheid in softwood, vessels, and fibers in hardwood (Figure 9). Plant cells contain a cell wall and lumen with a length of up to 7 mm and 10–70 μm in diameter. Pulping is a process of separating plant cells into individual fibers. After bleaching, fibers mostly contain cellulose and are thus also called cellulose fibers, which also refer to spun filaments of cellulose derivatives and regenerated cellulose, depending on the context in which it is used. Cellulose is macromolecules of glucose monomers, organized into hierarchical structures of elemental fibrils consisting of alternate amorphous and crystalline regions, microfibrils of aggregated elemental fibrils, mainly existing in the S1, S2, and S3 layers of a cell wall. Cell walls can be broken down, and various-sized components can be separated and harvested. Three representative sizes are cellulose fibrils (>100 nm in diameter, >200 μm in length) (Figure 9f), cellulose nanofibrils (CNFs) (5–100 nm in diameter, 0.5–200 μm in length) (Figure 9g), and cellulose nanocrystals (CNCs) (5–70 nm in diameter, <500 nm in length) (Figure 9h) [73]. Both CNFs and CNCs are also called nanocellulose.

### 4.2. Impact of Particle Morphology on Pore Size and Porosity of Membrane Materials

**Figure 10:**
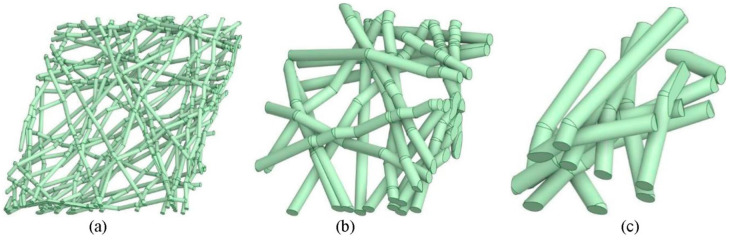
The relationship between fiber diameter and pore size in nonwoven membranes with the same porosity produced from a range of diameter sizes: (**a**) 0.1 μm diameter fibers, (**b**) 1.0 μm diameter fibers, and (**c**) 2.0 μm diameter fibers [78]. Pore sizes are tuned by constituent fiber diameters and the thickness of the membrane.

There are three types of pores in membranes made from fibers: inter-particle pores, fiber lumens, and pores inside cell wall material. The latter two pore systems are decided by the nature of the fibers. The porosity and separation properties are mainly decided by inter-particle pores, which are simply referred to as pores. A nonwoven membrane of fibers can be modeled as a stack of many thin sheets with the same pore size. Within a sheet, the diameter of fibers, fibrils, and crystals decides pore size and distribution, but adding one layer on a stack of sheets will reduce the pore size by half. So, the thicker the membrane, the smaller its pore size. Figure 10 illustrates three digital twin replica geometry of air filters of varied diameter sizes of cellulose particles. When the porosity is maintained, the effective mean pore size decreases with a decreasing fiber diameter. Generally, the mean pore size is directly proportional to the fiber diameter in nonwoven fiber membranes. At the same thickness of membrane, the air filtration efficiency and airflow resistance decrease exponentially with the fiber diameter but selectivity increases [78]. Generally, smaller diameter fibers lead to a higher number of pores and a smaller average pore size. 

Fiber lengths should have less effect on pore sizes than fiber cross-sectional dimensions. Longer fibers generally possess more kinks and orient themselves into a less compact form during formation and drying than short fibers, resulting in a larger pore size. Thick and stiff fibers can resist larger capillary action during drying, resulting in less fiber collapse and consolidation. Generally, fibers with a higher degree of polymerization are stronger. For example, cellulose fibrils with a degree of polymerization of 410 exhibits a membrane porosity of 20%, while fibrils with 1100 show a porosity of 28% [79].

### 4.3. Structure of Membrane Materials Made from Cellulose Particles

**Figure 11:**
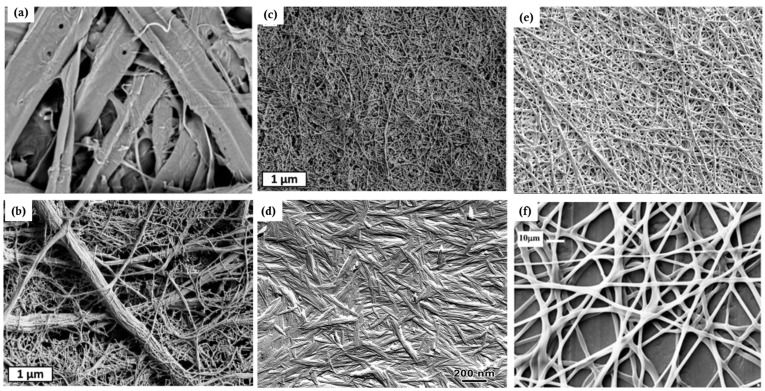
SEM morphology of membranes made from cellulose fibers (**a**) [83], fibrils (**b**), nanofibrils (**c**) [84], nanocrystals (**d**) [73], electrospun nanofibers of cellulose acetate (**e**) [85], and regenerated cellulose (**f**) [86].

Figure 11 shows the morphology of membrane materials made from various cellulose particles. Cellulose fibers originate from wood cells, which vary in size from species to species. The pore sizes of cellulose fiber paper were found to be 1–5 µm for the base layer and 50–500 nm for the coating pores [80]. The coating of a glassy paper has an average pore size of about 180 nm, and an estimated porosity and permeability of 34 percent and 0.09 mDarcy, respectively [81]. A base paper was estimated to have a pore size from 3 to 3.5 µm and 50–130 nm for the coating and a porosity of 32 to 44 percent for the base paper and 20 to 36 percent for the coating [82]. Hence, cellulose fiber paper can be considered microfiltration membranes, and its pigmented coatings fall into upper ultrafiltration and lower microfiltration.

Electrospinning is a versatile technique that can generate a wide range of fiber diameters depending on the process parameters and solution properties. Cellulose and its derivatives can be dissolved into solvents. The solutions can be spun into fibers with average diameters from 50 to 900 nm [78], which can form membranes of randomly distributed fibers (Figure 11e,f). The term nanofiber is usually used to designate those with diameters from 1–100 nm, while the term microfiber refers to those with diameters ranging from about 100 nm to 10 µm. In adjusting the electrospinning parameters (e.g., flow rate, applied voltage, coagulation conditions) and formulations of cellulose solutions, the surface chemistry and morphology, along with other physiochemical characteristics such as tensile strength of fibers, the swelling property, porosity, permeance, and adsorption, can be finely tuned for a specific pore size and distribution either as the support layer or selective layer. These materials have been mostly evaluated in labs without wide commercialization.

Decreasing the fiber diameter and increasing the fiber thickness can finely tune the pore size and topology of pore networks and hence affect the selectivity and permeance. CNFs with diameters in the range of 3–6 nm can produce a thin membrane barrier layer of 100 nm with a mean pore size on the order of 20 nm [87]. The obtained nanofibers have a uniform diameter of 7.5 ± 2.5 nm, thickness down to 23 nm, and pore sizes ranging from 2.5 to 12 nm, as well as a ferritin molecules rejection rate of 94% [88]. At 20 g m^−2^ (around 20 μm), a CNC membrane has an average pore size of 2.4 nm and that of a tetramethylpiperidine-1-oxyl (TEMPO)-oxidized CNF membrane is 19 nm with the water permeance for both at ~4 L m^−2^ h^−1^ MPa^−1^ [89]. CNCs are the smallest particles in size and form the thick, dense structure of membranes (Figure 11d) with pores of free volume less than 1 nm in diameter [90], which can be used to regulate gases and moisture in air. These pore sizes correspond to the ultrafiltration range. The sizes of the pores vary from micrometers to sub-nanometers for other membranes made from cellulose particles between cellulose fibers and CNCs.

### 4.4. Processing–Structure Relationship of Cellulose Particle-Based Materials

Papermaking or medium casting is performed to prepare membranes made from slurries, suspensions, or dispersions of cellulose particles. The dispersion is vacuum-filtered into a wet gel cake, followed by drying. The structure, pore size, distribution, and interconnectivity depend on the nature of the cellulose particles, medium, and drying method. The pore size measured via mercury intrusion porosimetry can range from 10 nm to 200 μm. The high surface tension of water collapses the pores between fibers during conventional drying, leading to a denser film structure [91]. To reduce the capillary forces and generate a larger pore structure, solvent exchange from water to low-surface-tension solvents such as 2-propanol and octane is applied, after which the gel cake is slowly dried in ambient conditions. In contrast with denser films dried from water-saturated cakes, the corresponding porous membranes are obtained from drying the solvent-swollen cakes [84]. It has been reported that the solvent exchange of water with acetone, ethanol, and methanol can increase the membrane porosity to 40, 38, and 28 percent, respectively, compared with 19% in water [92]. At the same 50 g m^−2^ grammage, the cationic CNF membranes cast by water and ethanol, prepared via supercritical CO_2_ drying, or freeze drying, has porosities of 37, 46, 73, and 79 percent. Although the freeze drying has a porosity similar to the supercritical drying, it has a topology different to that of the pore network displaying the highest permeance of 476 L h^−1^ m^−2^ MPa^−1^, which is about an order of magnitude higher than those of the other three membranes [93]. Slow drying leads to lower porosity within the same drying method, while fast drying results in higher porosity due to rapid water evaporation. Constraint drying results in greater porosity than with unconstrained drying.

Additionally, chemical modifications of cellulose, such as amination, carboxylation, silylation, and thiolation, along with cross-linking and surface coating techniques, affect surface charge and reactivity and play a vital role in tailoring membrane porosity and absorptiveness [29]. For example, aminated CNF membranes are developed with a porosity of approximately 80% and an average pore size of 0.38 μm, exhibiting impressive adsorption capacities for heavy metal ions while maintaining high water permeation rates. Similarly, cross-linking with different agents improves membrane porosity. Soyekwo reported the development of an ultrathin cross-linked-PEI selective layer through interfacial polymerization on ultrafine cellulose nanofiber as an intermediary layer, resulting in improved porosity at 0.45 nm [30]. Moreover, surface coating methods can control the pore size of a nanofiber membrane. In this approach, a thin barrier layer with the desired pore size can be applied to the nanofibrous scaffold, effectively controlling the membrane’s overall pore size [31]. The membrane made from 2% CNC-reinforced electrospun nanofibers of a hydrophobic copolymer increase the tensile strength and modulus as well as the liquid entry pressure [94]. In summary, processing conditions can finely tune pore size, distribution, and surface functionalities.

### 4.5. Cellulose Particle-Based Membrane Structure Design

**Figure 12:**
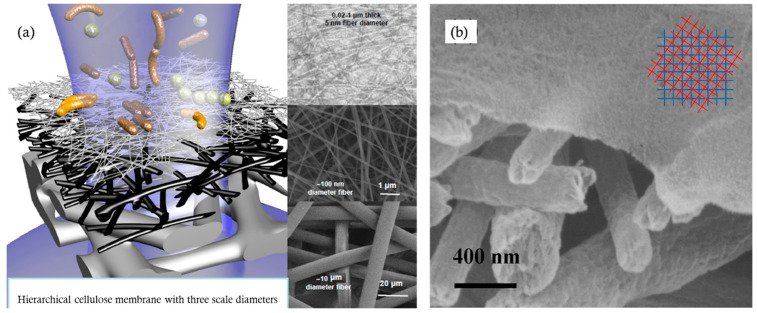
(**a**) Schematic membrane structure with hierarchical fiber diameters from microns to nanometers forming three-layered pores: the top thin ultrafiltration layer of nanoparticles, the middle microporous support layer of microfibers, and the bottom backing layer of fibers [92]. (**b**) A thin-film nanofibrous composite membrane with the top thin ultrafiltration layer (a maximum pore size of 55 nm at a thickness of 100–200 nm) of TEMPO-oxidized CNFs coated on a support layer of electrospun polyacrylonitrile microfibers [87]. The inset of the overlapping blue and red nets illustrates that the pore size decreases with increasing thickness.

Cellulose nanoparticles including CNCs and CNFs, with a size of 2–10 nm and fiber length of up to a few microns, are suitable for the construction of membranes with defined mean pore sizes for pressure-driven ultrafiltration and microfiltration in several ways: (1) self-standing membranes; (2) as a top selective layer supported by more porous substrates, as shown in Figure 12; (3) as a substrate layer providing a smooth surface for the interfacial polymerization of a thin dense polymer on it; (4) as a matrix reinforced by a polymer fiber scaffold; and (5) as a reinforced scaffold for a polymer matrix.

Conventionally, membranes are typically asymmetrical layered structures consisting of a thin selective layer on the top side, a finely porous support sublayer in the middle, and a coarsely nonwoven backing layer at the bottom. This construction provides structural strength to resist pressure and a smoother surface to support a selective layer that is thick enough to be selective but not so thick that it causes low permeation rates. The thin selective layer decreases the requirement of the driving force or pressure gradient and increases permeance; thus, the membrane may possess a higher permeation flux and can be operated at a lower pressure. Accordingly, cellulose particle-based membranes can be constructed with a thin layer of nanoscale CNCs or CNFs as the selective layer, cellulose microfibrils or electrospun cellulose nanofibers as the support interlayer, and cellulose fibers as the backing layer, as shown in Figure 12, but such a completely cellulose-based membrane has not been reported, while various composite three-layered structures have been studied.

**Figure 13:**
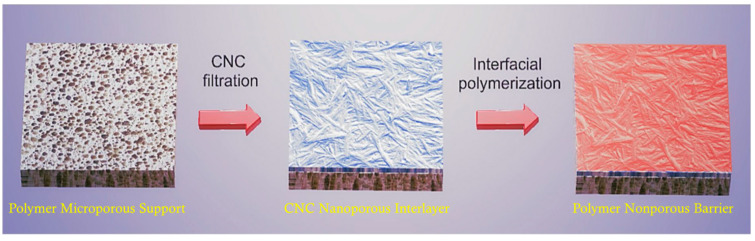
Schematic of the formation of a triple-layer composite membrane with CNC as the interlayer [5].

Using cellulose nanomaterials for barrier layer fabrication in the design of new filtration membranes has been reviewed, as shown in Figure 12 [92]. When TEMPO-oxidized CNFs are used as the barrier layer in an asymmetric three-layered nonwoven fibrous structure containing fibers of different diameters, the membranes exhibit a two- to ten-fold increase in the permeation flux over commercial membranes for the ultrafiltration of oil and water emulsions [95]. The thin-film nanoporous composite membrane has a pure water permeance several times higher than that of commercial polyacrylonitrile membranes because cellulose is more hydrophilic than polyacrylonitrile and has a nominal molecular weight cutoff of 2000 kDa at a rejection rate larger than 90%, which corresponds to a maximum pore size of 54.6 nm [87]. Serving as the interlayer between the coarse substrate support and thin selective layer, a nanocellulose layer can provide a smooth surface on which a thin dense selective polymer layer can be formed via interfacial polymerization to fabricate nonporous membranes for reverse osmosis [5,96], as shown in Figure 13, or a thin layer of graphene oxide is coated for dye removal [97]. The nanocellulose interlayer helps direct the water permeation due to its hydrophilic feature.

Based on the design shown in Figure 7a with a polymer network as the support, the two-layered nonwoven composite membranes, a layer of TEMPO-oxidized CNFs or CNCs (5–8 nm in diameter) infused into an electrospun polyacrylonitrile fibrous sheet (150 nm in fiber diameter) backed by a poly(ethylene terephthalate) nonwoven substrate (20 μm in diameter), exhibits a high water permeance and a high ability to remove bacteria (by size exclusion) and viruses (by adsorption) simultaneously [95,98]. It has been demonstrated that nanocellulose (CNFs and CNCs) can be embedded in a polymer matrix (e.g., cross-linked polyamide formed by interfacial polymerization) forming an interconnected fibrous scaffold, which acts as a directed water channel, leading to an increase in permeance without loss of selectivity [99]. CNC-reinforced chitosan membranes, with a thickness of 250 μm with pores of 13–17 nm, have a rejection rate of up to 99 percent for positively charged dyes [100].

### 4.6. CNF Membranes

CNFs have the potential to form filtration membranes because of their high aspect ratio to form porous structures with mechanical strength and hydrophilicity [101,102]. These membranes have an average pore size of 20 nm and feature more exposed functional groups [5,99,103]. CNFs form a colloidal structure in an aqueous solution. When CNFs come into contact with hydroxyl groups on neighboring nanofibrils during dewatering and drying, they intertwine and self-assemble into strong CNF membranes [104,105]. These integrated CNF membranes offer favorable strength characteristics and reduced permeability compared to other polymeric membranes (i.e., PET, PSf, etc.) of similar thickness, making them well-suited for various water filtration applications [106]. Furthermore, CNFs bring an extra dimension to filtration capabilities. Whether serving as the barrier layer or acting as a filler, CNFs can enhance filtration through adsorption. Typical CNFs contain negatively charged carboxylate groups with large surface-to-volume ratios, which makes them effective adsorbents for the removal of small, positively charged particles, molecules, and metal ions [87,106].

The literature extensively discusses the use of CNF-based self-standing membranes for water purification purposes [107,108,109]. In a study by Zhang et al., a self-standing CNF-based membrane was successfully developed using a dilute CNF solution and a combination of facile freeze-extraction methods, followed by the direct filtration of the nanofibril dispersion onto porous supports [88]. The CNFs with a consistent diameter of 7.5 ± 2.5 nm facilitated the formation of ultrathin nanoporous membranes. These membranes exhibited impressive flux rates of 1.14 and 3.96 × 10^4^ L m^−2^ h^−1^ bar^−1^ for pure water and acetone when the thickness of the membrane was 30 nm. In another study, free-standing and self-assembled hybrid membranes were reported, which combined TEMPO-oxidized CNFs (TOCNF) and graphene oxide (GO). These hybrid membranes showed promise in removing Cu (II) from water, along with exhibiting good recyclability and hydrolytic properties [110]. In this approach, TOCNF, which is rich in carboxyl groups, served not only as a binder and matrix but also as the primary functional component for adsorbing moieties containing positive charges within the biohybrids. The nanoGOs acted as connectors and nodes, combining with TOCNF to create a unique open porous network structure that significantly enhanced the water flux. The TOCNF membrane exhibited an impressive Cu (II) adsorption capacity of 114.1 mg g^−1^. The TOCNF + nanoGO hybrid membrane showed an adsorption capacity of 68.1 mg g^−1^, surpassing that of GO but falling short of TOCNF alone. This suggests that some functional groups, such as carboxyl groups on TOCNF are used for self-assembly rather than contributing significantly to the adsorption of Cu (II).

Moreover, CNFs have found application as barrier layers in thin-film nanofibrous composite (TFNC) membranes, which consist of multi-layered fibrous structures with a top barrier layer composed of CNFs or its nanocomposites [111]. These TFNC membranes have demonstrated exceptional effectiveness in improving the flux performance for microfiltration and ultrafiltration applications [22,98,99,112]. The inclusion of CNFs also imparts adsorption capabilities to these membranes. For example, in a study conducted by Chu et al., a TFNC membrane with a barrier layer made of TEMPO-oxidized CNFs, combined with an electrospun PAN/nonwoven PET substrate, exhibited a high adsorption efficiency of positively charged crystal violet dye molecules. Additionally, it displayed a favorable filtration rejection ratio against different bacteria and viruses at low pressure (19.3 kPa) [98]. The introduction of CNFs and microcrystalline cellulose into the electrospun PAN scaffold supported by a nonwoven PET substrate led to a reduction in the mean pore size of the composite membrane from 2.6 µm to a few hundred nanometers, enabling the removal of E. coli from water with a retention ratio of 99.9% [51]. Furthermore, CNFs, chitin nanofibers, and a blend of CNF/chitin nanofibers have been found to serve as suitable barrier layers in nanofiber membranes containing the electrospun PAN/nonwoven PET substrate. These three types of nanofiber barrier layers effectively decrease the pore size of the final membrane to 25, 27, and 14 nm, respectively, all of which are well suited for ultrafiltration to remove oil emulsions from water. The most efficient system demonstrates both a high flux performance (490 L m^−2^ h^−1^) and a high rejection ratio (99.6%). Additionally, the membrane featuring a barrier layer composed of a blend of CNF/chitin nanofibrils exhibits robustness, maintaining a consistent flux performance for 100 h, which is several times longer than that of a commercial PAN 10 membrane [112]. In another study, 2,3-dicarboxy cellulose nanofibrils with a width of 22 ± 4 and high anionic surface charge density served as a barrier layer on a porous PVDF membrane substrate, and the composite membrane successfully rejected molecules in the range of 35–45 kDa with an efficiency of 74–80% [113].

CNFs can also be employed in a polymeric matrix to create a nanocomposite barrier layer. For instance, interfacial polymerization was performed on CNF nanopaper using polyethylenimine and trimesoyl chloride to reduce the mean pore size of the membrane to less than 1 nm, making it suitable for nanofiltration applications [96]. This positively charged membrane exhibited a high permeation flux of 32.68 L m^−2^ h^−1^ bar^−1^ and decent rejection ratios against various salts (i.e., 65.3% for MgSO_4_, 89.7% for MgCl_2_, 43.6% for NaCl, and 39.1% for Na_2_SO_4_).

Additionally, CNF-based materials have found application in the field of water/oil separation. For example, Yin et al. reported the development of colorful superhydrophobic CNF-based membranes (CSNBMs) through a straightforward spraying method [114]. These membranes, treated with octadecyl trichlorosilane (OTS), pigment dispersions, and n-hexane, exhibit exceptional durability and high efficiency in both oil/water separation and oil spill cleanup. Thanks to their superhydrophobic and superoleophilic properties, as well as their inherent porous structure, the CSNBM demonstrates remarkable separation efficiency for various oil/water mixtures. Notably, it achieved the highest separation efficiency for n-hexane, exceeding 89%, and offers an impressive oil flux of 101.8 L m^−2^ h^−1^.

### 4.7. CNC Membranes

CNCs hold significant value in water membranes owing to their unique attributes, such as shorter length, heightened charge density, large surface area, good thermal stability, and impressive strength [25,115]. Notably, CNCs offer the capability to produce membranes with smaller and precisely controlled pore sizes, thanks to their inherent needle-like structure [116]. This feature proves especially advantageous when dealing with applications necessitating the filtration of minute particles or molecules. Additionally, CNCs typically exhibit a greater surface charge density due to their compact size and expanded surface area, enhancing the adsorption of charged particles and molecules from water [117]. Furthermore, they can be chemically modified easily to tailor their porosity and other properties [118]. Moreover, due to their hydrophilic nature and diminutive size, CNC-based membranes contribute to a reduction in fouling by preventing the accumulation of particles and contaminants on the membrane surface [119,120,121,122]. Furthermore, CNCs are renowned for their outstanding mechanical properties, encompassing a high tensile strength [119].

CNCs serve as crucial components in the construction of water filtration membranes, notably as functional layers. For example, CNCs have been employed as functional layers in layered cellulose nanocomposite membranes [123]. This synthesized membrane has pore structures in the microfiltration range (5.0–6.1 μm), resulting in remarkably high water permeability (900–4000 L h^−1^ m^−2^) at pressures below 1.5 bars. These membranes exhibit a tensile strength of 16 MPa in dry conditions and 0.2 MPa when wet. When they are used to treat model industry effluents containing metal ions (Ag^+^ and Cu^2+^/Fe^3+^/Fe^2+^), CNC-based membranes have demonstrated effective removal rates. The efficiency of removal varies depending on the functional groups of the CNCs that were employed, with phosphorylated CNC membranes exhibiting the highest removal rates.

**Figure 14:**
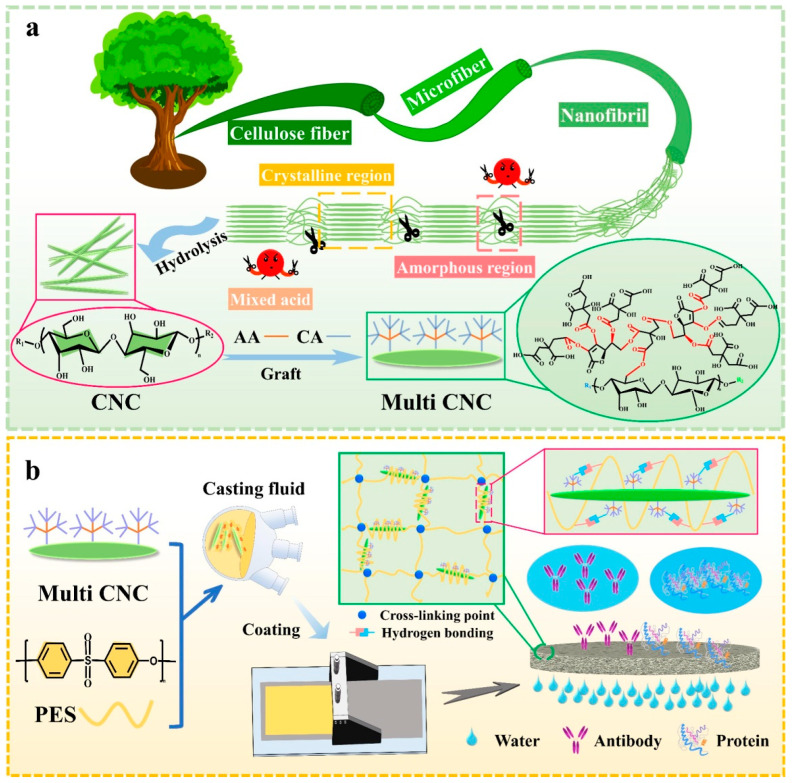
Diagram of muti-CNC/PES-based membranes adapted with permission from reference [125].

CNCs are also used to develop composite membranes with different organic polymers (such as PES, PVDF, PSF, etc.) [124,125]. For instance, in a study by Zheng et al., high-performance ultrafiltration membranes were fabricated by blending multi-branched nanocellulose (multi CNC) within a polyethersulfone (PES) matrix using anti-solvent-induced phase separation (NIPS) technology, as shown in Figure 14 [125]. This composite ultrafiltration membrane exhibits high hydrophilicity, a thicker skin layer, and a lower negative charge due to the presence of carboxyl (-COOH) groups in multi-CNCs. Consequently, this membrane shows a significantly higher water flux (962 L m^−2^ h^−1^) and an excellent Bovine serum albumin (BSA) rejection (96.4%) compared to the original PES membrane. In another study, CNC-PVDF membranes were developed for wastewater treatment, which significantly increases permeability (up to 206.9 L m^−2^ h^−1^) due to improved porosity, surface roughness, and hydrophilicity as compared to PVDF (9.8 L m^−2^ h^−1^) [124]. In a study conducted by Bai et al., a CNC/polyamide TFC was developed onto a PES membrane using interfacial polymerization [117]. When only 0.2 wt.% of CNC was added to the polyamide thin film, the pure water permeability under 0.25 MPa increased by 60%, while the rejection of NaCl rose from 16.19% to 22.7%. Even with an increased CNC content, the rejection of SO_4_^2−^ divalent salts remained consistently high at over 98%. Similarly, Asempour et al. developed a TFC reverse osmosis membrane by incorporating CNC into the polyamide active layer. This CNC/polyamide nanocomposite membrane proved effective in recovering water from synthetic brackish water. Importantly, the study revealed that the permeability of the membrane doubled to 63 L m^−2^ h^−1^ with no significant impact on salt rejection, which remained at 97.8%, even when a small amount (0.1 *w*/*v*%) of CNC was incorporated [126].

CNCs combined with other biomaterials to create sustainable membranes have also been investigated. For example, CNC/chitosan composite membranes have been prepared through freeze drying, compaction, and cross-linking using glutaraldehyde vapors. These membranes exhibit enhanced mechanical properties, a suitable pore diameter for ultrafiltration, and high efficiency in adsorbing positively-charged dyes, namely Victoria blue (98%), methyl violet (90%), and rhodamine 6 G (78%) [100].

In oil/water separation, CNC-based hybrid membranes have been developed with resistance to swelling. These hydrophilic membranes feature highly interconnected channels, ensuring a non-swell ability and maintaining mechanical and structural integrity. For instance, Wang et al. developed CNC/H-PAN/PEI/SiO_2_ hybrid membranes that showed high separation efficiencies, exceeding 94% for various emulsions, even after 20 filtration cycles. In this process, CNCs play a key role in improving the development of highly interconnected channels for improving hydrophilicity and an efficient non-swell ability [127].

### 4.8. Bacterial Nanocellulose (BNC) Membranes

BNC-based membranes are gaining recognition for water purification, characterized by their high purity, substantial aspect ratio, ultrafine structure, and exceptional resistance to biofilm formation [128,129,130]. One noteworthy advantage of BNC-based membranes is their uniform availability of hydroxyl and carboxyl functional groups, coupled with a substantial aspect ratio, which enhances their hydrophilicity and increases the water holding capacity, ensuring consistent water flow during filtration processes [130,131]. Additionally, BNC permits precise control over pore structure and size, owing to its highly consistent fiber diameter (ranging from 30 to 50 nm), making it highly adaptable for customizing membranes to meet specific filtration requirements [5,131,132]. Furthermore, the highly interwoven network structure of BNC, along with a high crystallinity index of up to 86.94%, significantly enhances the mechanical properties of BNC-based membranes [133,134].

Recently, self-supported membranes based on BNC have shown high efficiency in the removal of organic dyes and heavy metals from water. For instance, Ferreira-Neto et al. developed a porous BC/MoS_2_ hybrid aerogel membrane with a high surface area, preserved porosity, and tunable MoS_2_ interlayer spacing for in-flow water purification. This innovative membrane has demonstrated an exceptional performance in the simultaneous removal of methylene blue dye (96% removal within 120 min) and heavy metal ions (88% Cr^+6^ ions removal within 120 min) under UV–visible light, while also exhibiting excellent recyclability and photostability [135]. In another study conducted by Gholami et al., a polydopamine (PDA)/BNC membrane achieved a notable water flux of 57 L m^−2^ h^−1^ under a vacuum pressure of 0.7 bar. This membrane demonstrated an effective removal of heavy metal ions, including Pb^2+^ and Cd^2+^, as well as organic dyes, serving as surrogate markers for organic pollutants, such as rhodamine 6G (R6G), methylene blue, and methyl orange, across a pH range from 4 to 7 [136]. Furthermore, Yang et al. enhanced the performance of the PDA/BNC membrane by incorporating TiO_2_ nanoparticles, resulting in a photocatalytic (PDA/TiO_2_/BNC) thin film. This modified membrane displayed a rapid photocatalytic degradation of organic dyes, including methyl orange, methylene blue, and rhodamine B, achieving efficiencies exceeding 95% within 1 h [134].

Furthermore, BCN can be incorporated with other 2D nanomaterials (graphene) to improve the water filtration properties. In a study by Xu et al., BNC was combined with GO and palladium nanoparticles. This membrane exhibited excellent methylene orange degradation, reaching up to 99.3%. It maintained a steady water flux of 33.1 L m^−2^ h^−1^ for six hours under a pressure of 58 psi. The unique mass transport characteristics of the GO-based BNC membrane were attributed to nanochannels in the lamellar stacks, which improved water permeation [133]. Jiang et al. extended the capabilities of BNC membranes by incorporating reduced GO (rGO), resulting in an antifouling membrane [137]. This membrane exhibited a superior photothermal effect due to rGO, achieving complete bactericidal activity under light illumination. Importantly, it demonstrated resilience to high pressure, vigorous mechanical agitation, and various pH conditions. Comparative studies revealed that the rGO/BNC membrane outperformed commercial membranes, achieving a higher water flux of 52.6 L m^−2^ h^−1^ at 100 psi, compared to just 21.6 L m^−2^ h^−1^ for the commercial membrane at the same pressure [137].

### 4.9. Antifouling

In the literature, it is well reported that hydrophobic surfaces tend to attract proteins and bacteria through hydrophobic interactions. Consequently, there is a growing consensus on the importance of modifying membrane surfaces to enhance their hydrophilicity as a key strategy to combat fouling [138].

Nanocellulose stands out due to its inherent hydrophilic nature, which naturally lowers its susceptibility to fouling. Furthermore, nanocellulose carries an electric charge that generates repulsive forces between the membrane surface and colloids bearing a similar charge, effectively acting as a barrier to further mitigate fouling [139]. For example, when TEMPO-oxidized CNFs with carboxylate functional groups are used as a barrier layer on an electrospun polyacrylonitrile (PAN) support layer, the resulting TFNC membrane achieve an impressive 97% removal of BSA, serving as a model protein foulant [140]. Similar outcomes have been observed in other studies where TEMPO-oxidized CNFs and PVA were coated onto a polyethersulfone (PES) membrane. These coatings exhibited affinity for positively charged dyes while demonstrating effective antifouling properties.

Individual filtration tests have demonstrated a 97% removal of Victoria blue [141]. Similarly, other hydrophilic polymers may also reduce the adsorption of proteins and cells to some extent. However, the primary antifouling effectiveness of any polymer largely hinges on its steric repulsions and surface hydration capabilities. In this context, NC offers a distinct advantage as it can be easily modified in various ways to tailor its antifouling properties [138].

### 4.10. Factors Affecting Mechanical Strength

Nanocellulose is highly effective in enhancing mechanical properties due to its exceptional characteristics. In particular, the dry membrane strength is highly improved due to the high aspect ratio, nanoscale dimensions, and abundant surface hydroxyl groups resulting from nanocellulose forming strong intermolecular bonds within the membrane matrix [142]. This reinforcement effect results in an increased tensile strength, Young’s modulus, and overall structural integrity of the membrane. Additionally, its capacity to decrease membrane porosity while preserving uniformity guarantees a consistent and resilient mechanical performance [143].

For the nanocellulose membrane, to enhance its wet strength, various commercially available wet-strength resins, such as urea–formaldehyde, melamine resins, alkaline-curing polymeric amidoamine-epichlorohydrin resins, and glyoxalated polyacrylamide resins, are commonly employed, while urea–formaldehyde, melamine, and polymeric amidoamine-based resins are dominated [144]. Recently, secondary amines with 3-hydroxy-azetidinium rings were reported to enhance the wet strength of nanopapers made of TEMPO-oxidized nanocellulose [145]. The reactivity of the azetidinium ring with the carboxyl group of nanocellulose plays a pivotal role in achieving a high wet strength. In addition, various bio-based wet-strength resins, including chitosan and water-soluble polysaccharides, with their chemistry relying on interactions, whether physical or chemical, between the functional groups on the resin and those on the nanocellulose, have been reported [146,147,148]. Likewise, various cross-linking agents have been documented to improve the wet strength of nanocellulose [146]. These agents include citric acid, low-molar-mass polyethyleneimine (PEI), inorganic salts like calcium chloride and sodium trimetaphosphate, glutaraldehyde, CaCl_2_ treatment, and glycidyl trimethyl ammonium chloride [22,146,149,150]. Typically, the chemical interactions between nanocellulose and these cross-linking agents involve covalent or ionic bonding mechanisms [100,149]. It has also been reported that counter-ion interactions also improve the wet strength of nanocellulose membranes [144].

Hot pressing at specific temperatures and extended durations can enhance the wet strength of NC-based membranes [95]. This improvement arises due to irreversible hornification [144,151,152]. For instance, a study by Osterberg et al. demonstrated that prolonging the hot pressing time from 0.5 to 2 h results in an increase in tensile strength from approximately 120 to about 225 MPa at a similar strain-to-failure rate of around 6% [153]. Additionally, hot pressing is commonly employed to create chemically resistant CNF films. However, prolonged exposure to heat while the membrane is in a wet state can lead to reduced porosity in CNF membranes and an increase in Young’s modulus [154].

## 5. Summary and Outlook

Cellulose is a biopolymer and exists in plant cell walls or is synthesized by bacteria. Natural cellulose can be extracted through pulping or the chemical/mechanical breakdown of cell walls into cellulose fibers or pulp, microfibrils, nanofibrils, and nanocrystals; dissolved in solvents into separated macromolecules; or derivatized into cellulose derivatives. These particles and macromolecules can be assembled into neat materials with various shapes, sizes, pore sizes, and distributions, as well as physical, chemical, and mechanical properties, which determine the membrane performance in terms of permeance and selectivity. They can also form composites with other materials, such as cellulose particle-reinforced polymeric materials, inorganic reinforced cellulose, and biopolymer composites of cellulose, starch, chitin, and proteins. Cellulose acetates can be used to make membranes with a wide range of pore structures from microfiltration to nanofiltration, but they currently do not perform as well as petroleum-based polymeric thin-film composite membranes for reverse osmosis filtration. Regenerated cellulose membranes are widely available for dialysis and can be microporous to nanoporous. Paper filter membranes made from natural cellulose fibers are staples in labs, offices, and homes for the removal of air or water-suspended pollutants and contaminants. Nanocellulose, including cellulose nanocrystals, nanofibrils, and electrospun cellulose nanofibers, shows remarkable potential for fabricating membranes and is the focus of current research and developments across the world. Still, it has yet to be commercialized for a specific application.

The field of membrane technology has witnessed a surge in interest in cellulose-based membranes in recent years. These membranes hold immense promise for various environmental applications. These include effectively removing pollutants and contaminants from wastewater streams, efficiently capturing dust particles in air filtration systems, and sustainably separating desired gases from unwanted components in gas mixtures. However, unlike petroleum-based thermoplastic polymers that can be melted and molded, cellulose itself is not readily processable in this way. Solution and suspension processing methods are two viable approaches for producing cellulose membranes. There are many environmental and technical challenges associated with them.

The traditional derivatizing solution process (viscose) for producing cellophane falls under the solution processing umbrella. In this method, cellulose undergoes a series of chemical reactions to create a viscose solution. This solution can then be shaped into a membrane film and regenerated back to cellulose through controlled chemical treatments. However, chemical treatments cause serious environmental problems, hinder workplace safety [155], and induce irreversible damage to the cellulose structure, deteriorating its excellent resistance to acid, alkali, and organic solvents [39,41].

Direct cellulose solvents involve dissolving cellulose in specialized solvents that can break down its strong intermolecular bonds. Once dissolved, the cellulose solution can be cast into a desired form (membrane) and solidified through solvent evaporation or anti-solvent precipitation. The membrane, prepared by directly dissolving pure cellulose, can keep cellulose’s native characteristics, including remarkable hydrophilic properties and good solvent resistance. Among cellulose solvents, NMMO and ionic liquids have great potential and advantages over traditional derivatizing solution methods as they can be efficiently reused as green solvents [156,157]. Using green solvents for cellulose opens new avenues for cellulose membrane development.

While green solvent processing offers a viable approach, cellulose dissolution and regeneration are not low-cost processes. Alternative methods, such as using cellulose particles in suspensions, have also been explored. Cellulose particles can be produced mechanically or by combining mechanical, chemical, and biological methods, potentially preserving natural cellulose characteristics and minimizing environmental impacts. Conventional pulping and papermaking technology can use cellulose particle suspensions to fabricate membranes. However, this technique comes with its own set of drawbacks. Cellulose particle suspensions are unstable over time, leading to potential aggregation and uneven particle distribution. Membranes fabricated from cellulose particle suspensions can exhibit uneven density throughout the structure, potentially compromising their performance and functionality. Suspensions are limited in terms of the maximum achievable cellulose particle concentration. This translates to a lower production efficiency for cellulose membranes and larger energy consumption for dewatering and drying. For ultrafiltration and nanofiltration membranes, cellulose particles must be refined into nanoscales. Production often requires expensive equipment, intensive energy consumption, and potentially hazardous chemical reagents.

Cellulose-based membranes offer a sustainable and attractive alternative for various applications. However, the processing methods employed significantly influence the final membrane characteristics. While solution processing offers a well-established approach, further research is needed to address the limitations associated with suspension processing, particularly regarding stability, uniformity, and cost-effectiveness.

Cellulose offers many interesting opportunities in filtration technology with unprecedented chemical and structural tunability, allowing rational design for flexible capacities and specific applications. With increasing concerns about polymer pollution and climate change, there is a need to research and develop new science and technology on cellulose membranes to replace synthetic polymer membranes without compromising separation efficiency. We envision a cellulose-based society where membrane structures are based entirely on cellulosic components, and cellulose will be an ideal inexpensive and sustainable platform that can be utilized to purify water, capture climate-warming gases, and recover water and heat from waste discharges and exhaust air streams.

## Figures and Tables

**Table 1:** Particle size range and terminology.

Size Range	Terminology
Nanoscale 1–100 nm	nanoparticles, nanomaterial, electrospun nanofibers, nanofibrils, nanocrystals, nanoporous, nanopores, nanofiltration (1–2 nm), ultrafiltration (2–100 nm)
Microscale 100 nm–10 µm	microfibrils, microcrystals, microfibers, electrospun microfibers, fibrils, crystallites, microporous, micropores
Macroscale >10 µm	fibers, electrospun fibers, crystals, microporous, macropores

**Table 2:** Kinetic diameters and critical temperatures of common gases.

	H_2_O	H_2_	CO_2_	O_2_	H_2_S	N_2_	CO	CH_4_	C_2_H_6_
Kinetic diameter (nm) *	0.265	0.289	0.33	0.346	0.36	0.364	0.376	0.38	0.43
Critical temperature (K) **	647.1	33.2	304.2	154.6	373.5	126.2	133.2	190.6	305.3

* [17], ** [18].

## Data Availability

Not applicable.
